# Supplementary figures and images for: Allosteric inhibition of trypanosomatid pyruvate kinases by a camelid single-domain antibody
Source: eLife. 2025 Mar 31;13:RP100066. doi: 10.7554/eLife.100066 (PMC11957543; doi:10.7554/eLife.100066)

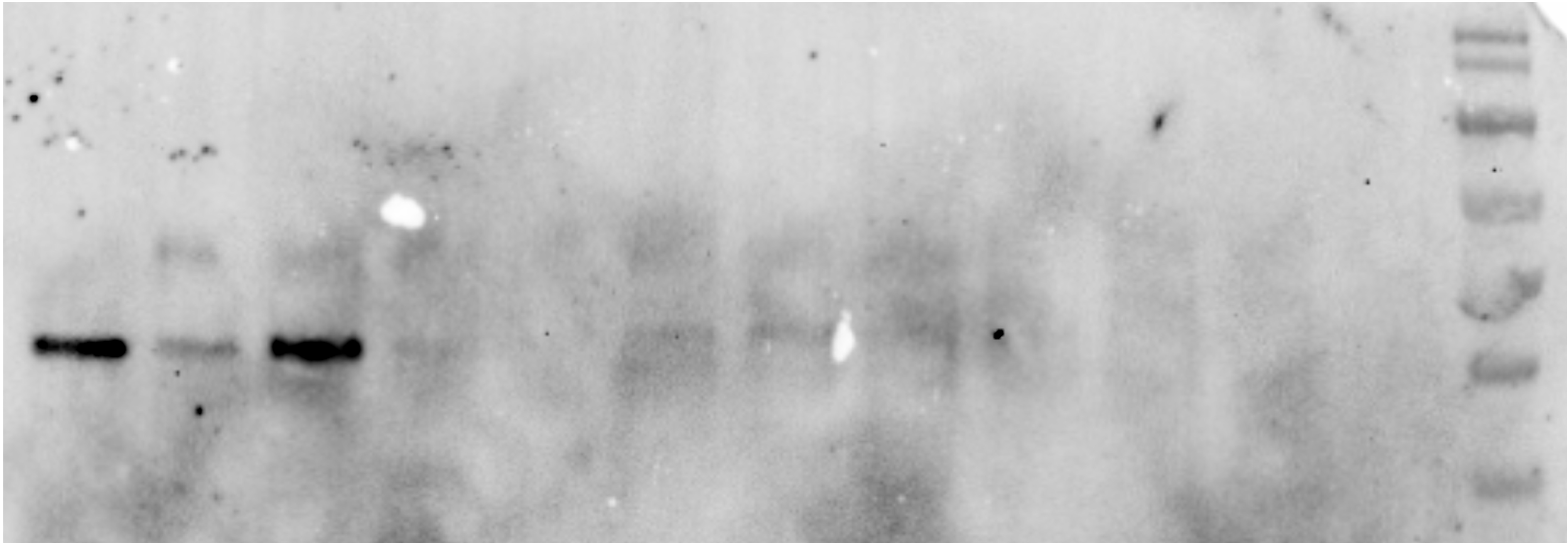

Supplement: Figure 7—figure supplement 1—source data 1. [file elife-100066-fig7-figsupp1-data1.zip › Western_blot1.jpg]

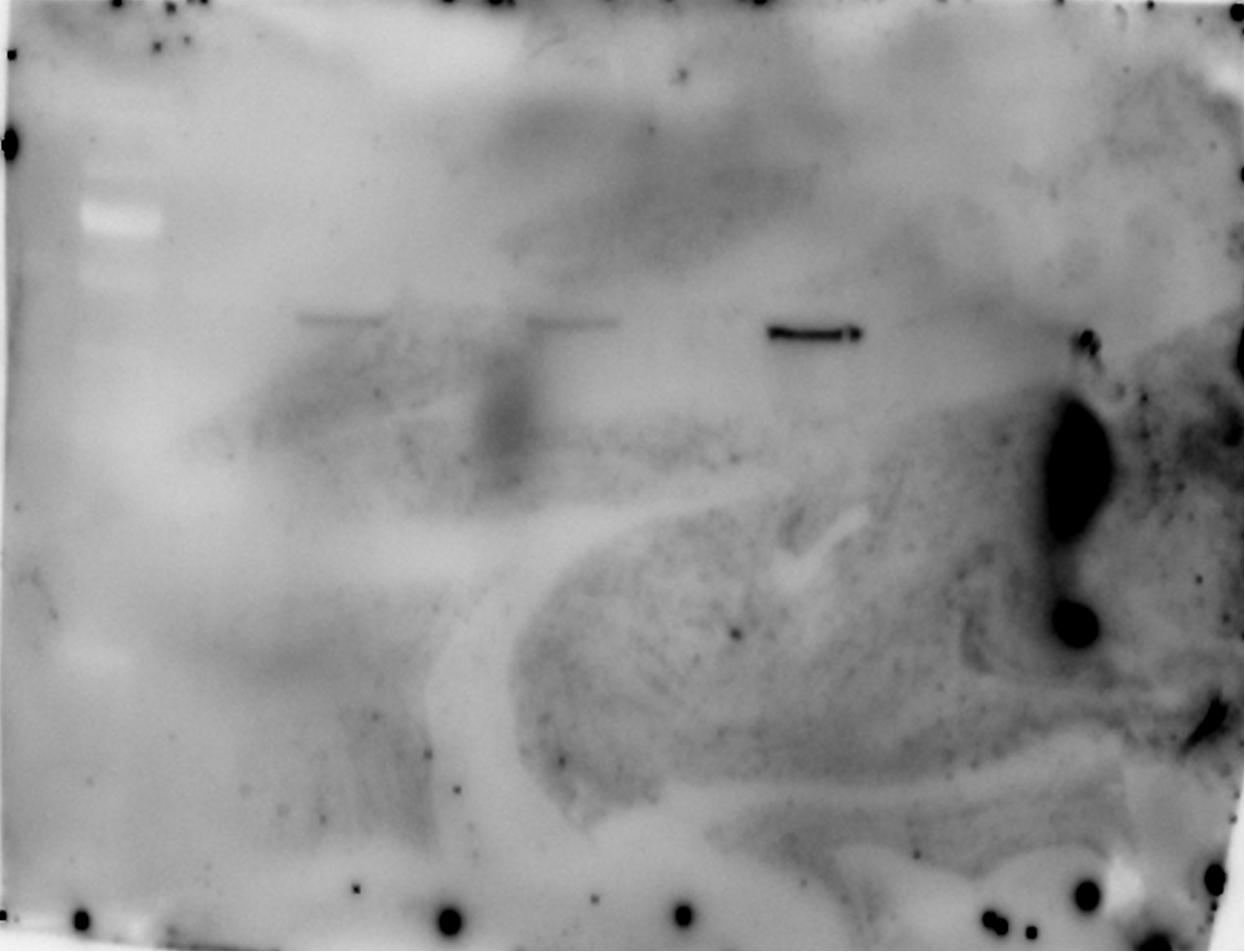

Supplement: Figure 7—figure supplement 1—source data 1. [file elife-100066-fig7-figsupp1-data1.zip › Western_blot2.jpg]

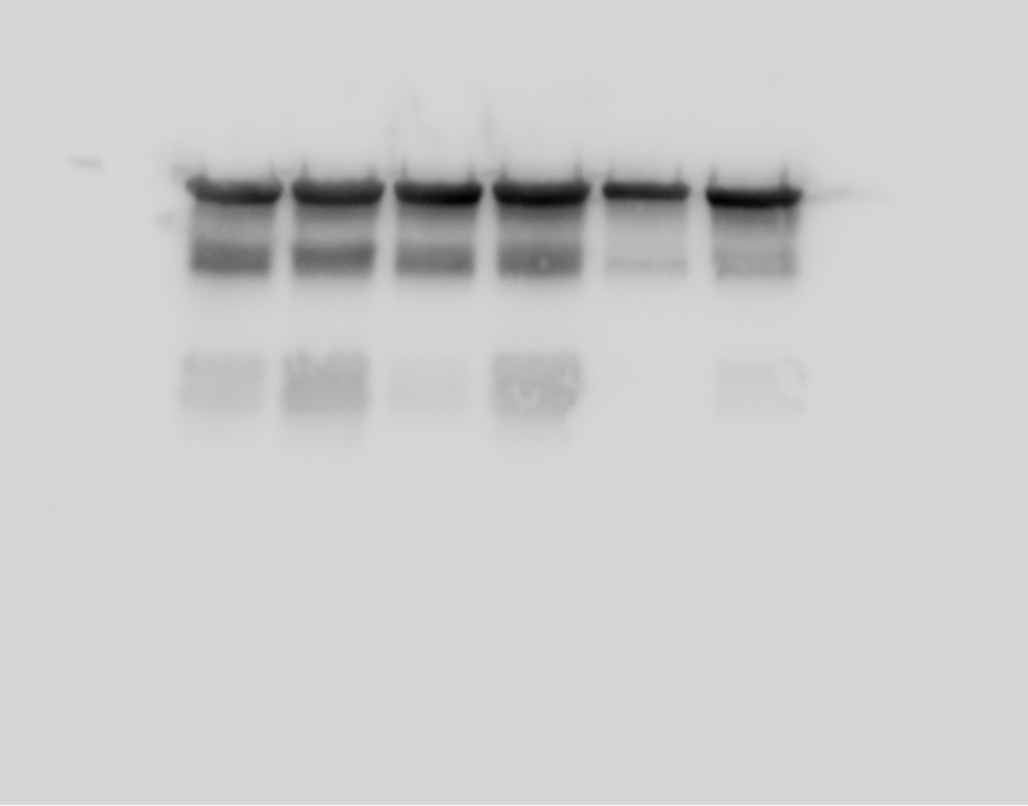

Supplement: Figure 7—figure supplement 1—source data 1. [file elife-100066-fig7-figsupp1-data1.zip › Western_blot3.jpg]

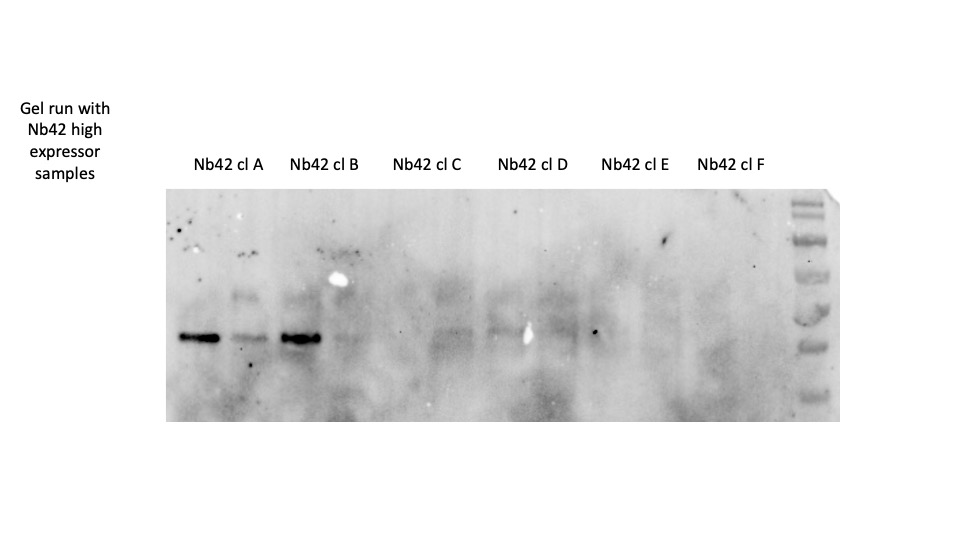

Supplement: Figure 7—figure supplement 1—source data 2. [file elife-100066-fig7-figsupp1-data2.zip › Western_blot1_with_annotations.jpg]

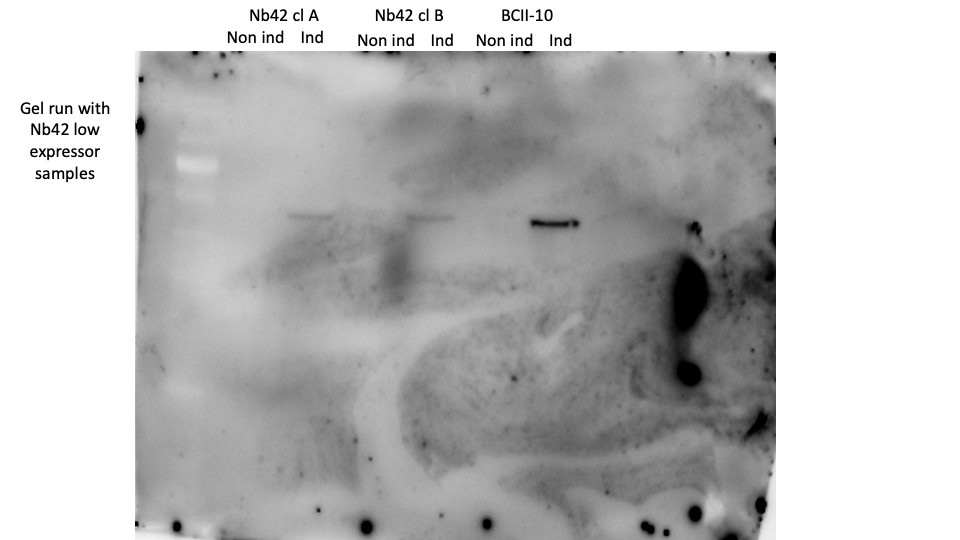

Supplement: Figure 7—figure supplement 1—source data 2. [file elife-100066-fig7-figsupp1-data2.zip › Western_blot2_wth_annotations.jpg]

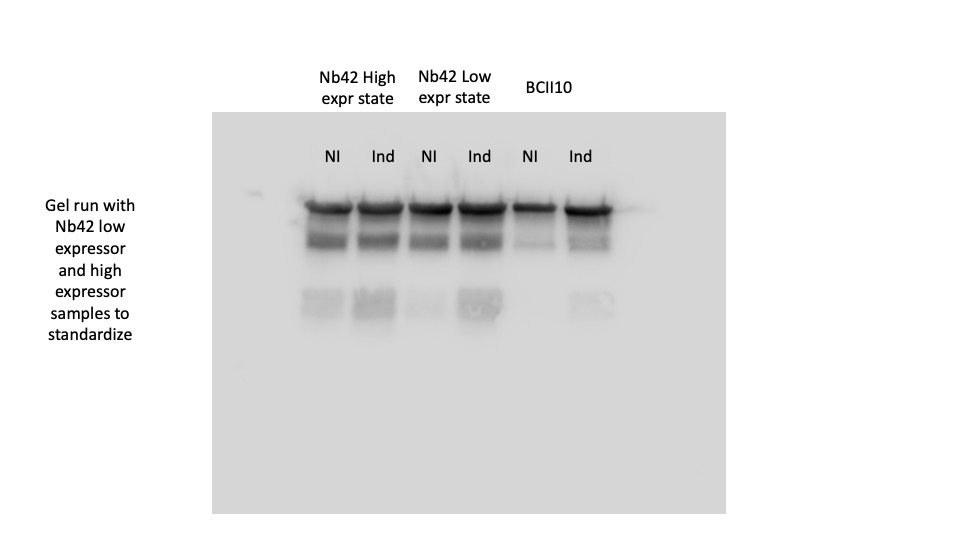

Supplement: Figure 7—figure supplement 1—source data 2. [file elife-100066-fig7-figsupp1-data2.zip › Western_blot3_with_annotations.jpg]
